# The effects of GLP-1 agonists on HbA1c and insulin dose among patients with type 1 diabetes

**DOI:** 10.3389/fendo.2025.1550938

**Published:** 2025-08-07

**Authors:** Amirah Alhowiti, Hyder Mirghani

**Affiliations:** 1 Department of Family and Community Medicine, Faculty of Medicine, University of Tabuk, Tabuk City, Saudi Arabia; 2 Department of Internal Medicine, Faculty of Medicine, University of Tabuk, Tabuk City, Saudi Arabia

**Keywords:** glucagon-like peptide agonists, type 1 diabetes, HbA1c, insulin dose, insulin reduction

## Abstract

Type 1 diabetes mellitus (T1DM) is a common chronic disease, and there is a rising trend globally; insulin is the mainstay therapy. Despite improvements in insulin delivery and monitoring, a significant percentage of patients still fail to reach glycemic targets. There is an increasing interest in using glucagon-like receptor agonists as adjuvant therapy. A high risk of bias limits meta-analysis on the effectiveness of GLP-1 agonists. This meta-analysis aimed to assess the effects of GLP-1 agonists on HbA1c and total daily insulin dose in T1DM. We searched PubMed, Cochrane Library, and Google Scholar for articles investigating the effects of GLP-1 agonists on the HbA1_c_ and total daily insulin dose without limitation to the publication date. The keywords used were GLP-1 agonists, liraglutide, albiglutide, exenatide, glycated hemoglobin, HbA1c, insulin dose, and glycemic control. Out of the 713 articles retrieved, 21 full texts were screened, and 10 trials were included in the meta-analysis. GLP-1 agonists are more effective than placebo in HbA1c reduction, *Z* = 5.27, SMD, 0.23, 95% confidence interval (CI), 0.14–0.32, with 1.2 mg and 1.8 mg more effective than 0.6 mg, SMD, −0.87, 95% CI, −1.60 to 0.13, and SMD, −0.79, 95% CI, −1.18 to 0.41, respectively. GLP-1 agonists reduce total daily insulin dose SMD, 2.21, 95% CI, 0.43–3.98 with no significant differences between different doses. GLP-1 agonists were effective in HbA1c and total daily insulin reduction among patients with T1DM. Liraglutide 1.2 mg may be more beneficial; further randomized trials focusing on different doses of GLP-1 agonists and hypoglycemia risk are recommended.

## Introduction

Type 1 diabetes mellitus (T1DM) constitutes 2%–5% of diabetes, and the disease is steadily rising worldwide; insulin replacement is the mature cornerstone therapeutic option. Despite the advances in insulin technology and the development of smart insulin preparation, patients with T1DM are not meeting the glycemic standard. A study conducted in Europe showed that 49.9%–72.4% of patients with T1DM were not achieving the glycemic target (1). Patients with T1DM are prone to both hyperglycemia and poor glycemic control on one hand and hypoglycemia on the other hand. In addition, patients with T1DM are at high risk of cardiac and renal complications (2, 3). Furthermore, obesity and overweight are increasingly observed among patients with type 1 diabetes mellitus (4).

Insulin therapy is the standard of care in patients with T1DM at the expense of increasing weight, hypoglycemia, and lack of cardiovascular protection (5–7). Therefore, add-on therapies with cardiac and renal protection and weight reduction properties are a great wish for diabetics and doctors. Glucagon-like peptide agonists–1 (GLP-1) with their effects on α and β cell preservation are good options for patients with T1DM, in particular those with detectable C-peptide and those with obesity/overweight (8). Liraglutide in combination with anti-interleukin (IL)–21 antibody preserved β-cell function with a good safety profile in a recent study (9).

The field of diabetes is rapidly evolving, and three stages of T1DM have been recognized. The stages are stage 1, characterized by multiple autoantibodies on two occasions; stage 2 includes autoantibodies and prediabetes; and stage 3, overt diabetes, according to the American Diabetes Association (10). Since the discovery of insulin >100 years ago, patients with type 1 diabetes have been waiting for a landscape change in their management.

Glucagon peptide-1 receptor agonists were approved for type 2 diabetes treatment (twice-daily exenatide) in 2005, with many long-acting classes developed in the following years. Semaglutide once-daily subcutaneous injection was approved in 2017 and 2021 for glycemic control and weight reduction, respectively. Oral semaglutide is now approved for type 2 diabetes and was shown to be cost-effective (11–13).

Adjuvant therapy, including GLP-1-like receptor agonists and sodium-glucose cotransporter-2 inhibitors, is still used sparingly (14). GLP-1-like receptor agonists’ action through glucagon suppression, delaying gastric emptying, and appetite suppression is promising in T1DM. The benefits are postprandial blood glucose reduction, lower insulin dose, weight loss, and cardiorenal protection. However, hypoglycemia, extra injections, and costs are major limitations. Therefore, proper patient selection is needed (15).

T1DM is linked to long-term complications, decreased quality of life, high mortality, and significant economic burden. HbA1c reduction is an important aspect of diabetes care to reduce microvascular complications, including retinopathy, neuropathy, and nephropathy (16).

T1DM is usually associated with other autoimmune diseases, including Addison’s disease and autoimmune thyroid disease. Importantly, T1DM with additional autoimmunity is associated with both hypoglycemia and hyperglycemia. In addition, patients with Hashimoto’s thyroiditis and Addison’s disease require higher doses of insulin compared to isolated T1DM (17). Therefore, investigating adjuvant therapy that can positively affect autoimmunity and weight is highly relevant. Because of the above, we assessed the effect of GLP-1 agonists on HbA1c and total daily insulin dose in T1DM.

Meta-analyses on the benefits of GLP-1 agonists on glycemic control, weight, and insulin dose reduction are scarce and have big limitations. Wang et al. published a meta-analysis that included seven trials and found lower glycated hemoglobin, total daily insulin dose, and body weight among patients with additional GLP-1 agonists compared to their counterparts (18). His findings were supported by Kim et al. who included six trials (four on liraglutide) and found weight reduction and better HbA1c levels (19), Karakasis et al. (20) included six trials and supported Kim et al. findings. Tan et al. included 11 trials and found similar observations (21). However, the authors included studies published by the same authors, and some included studies are not found in the references list.

## Subjects and methods

This systematic review and meta-analysis were conducted during November and December 2024 to assess glucagon-like receptors agonists (GLP-1 agonists) effects on the glycated hemoglobin (HbA1_c_), and total daily insulin dose among patients with T1DM. The study was conducted according to the PRISMA Guidelines.

### Eligibility criteria

#### Study type

The studies were included if they were randomized control trials and conducted on humans with a minimum duration of 4 weeks. The studies must compare the effects of GLP-1 agonists versus placebo on the glycated hemoglobin (HbA1_c_) and total daily insulin dose.

### Exclusion criteria

Retrospective, prospective studies, cross-sectional, case-control studies, opinions, editorials, commentaries, and protocols were excluded. Studies conducted in type 2 diabetes, and trials comparing other drugs (DPP4 inhibitors, SGLT-2 inhibitors, and monoclonal antibodies) with GLP-1 were eliminated from the study. Studies on animals, and study duration < 4 weeks were not included.

### Participants

Patients with T1DM, have no limitations regarding age, duration of diabetes, and C-peptide levels.

### Interventions and controls

The intervention was the use of GLP-1 agonists versus placebo.

### The outcomes measures

The outcome measures were the effects of GLP-1 agonists versus placebo on HbA1c and total daily insulin dose.

### Literature search

**Figure 1:**
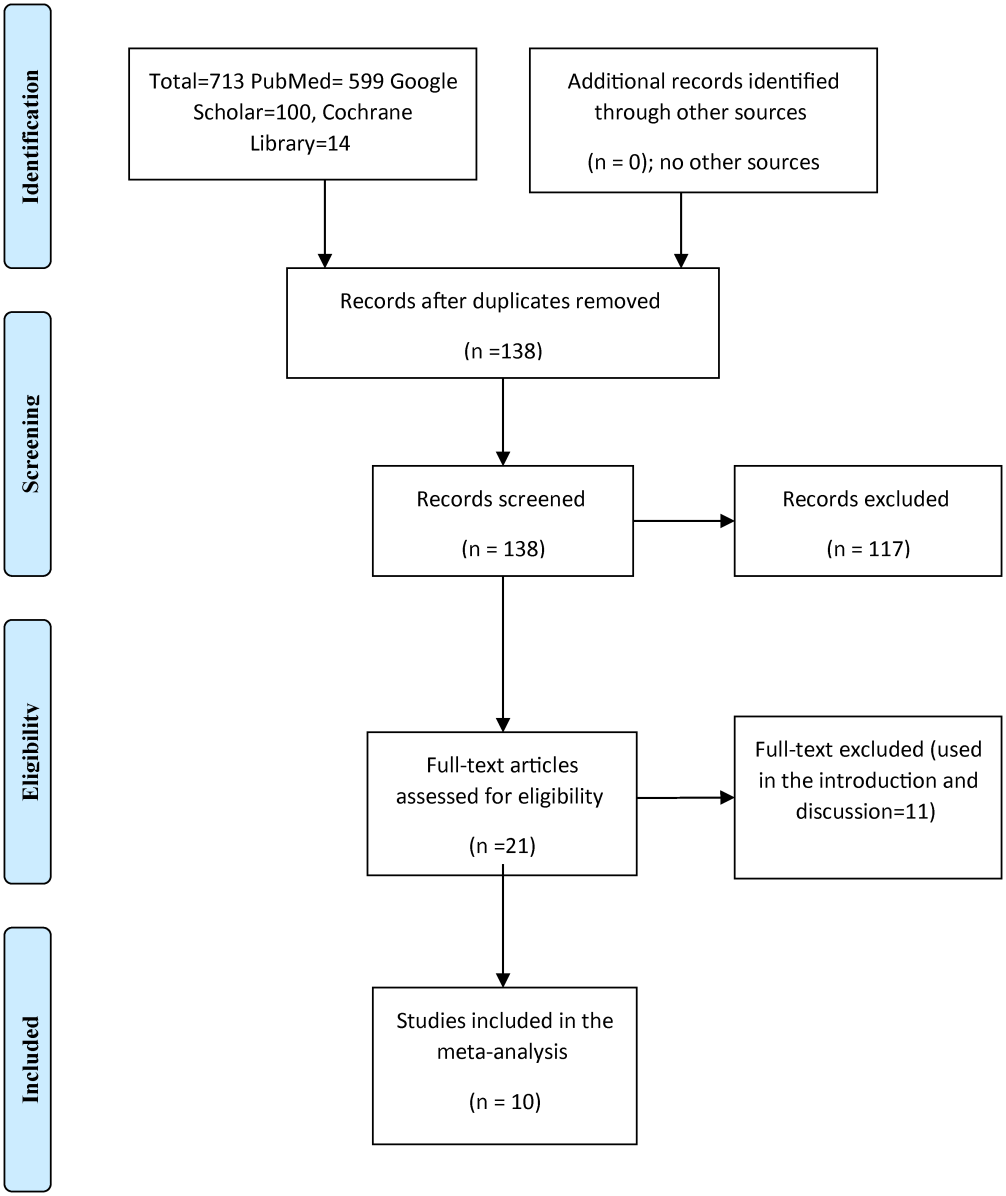
Literature search to retrieve articles assessing the effects of GLP-1 agonists on HbA1c, and insulin dose among patients with type 1 diabetes. (The PRISMA chart).

We searched Google Scholar, PubMed, and Cochrane Library for articles published in English and evaluated the effects of the glycated hemoglobin (HbA1_c_) and total daily insulin dose. The terms used were GLP-1 agonists, liraglutide, albiglutide, exenatide, glycated hemoglobin, HbA1c, insulin dose, and glycemic control. The search engine was limited to articles published in the English language with no limitation regarding the date of publication. Out of the 713 articles retrieved, 138 remained after the removal of duplication, of them 21 full texts were screened, and nine trials were included in the meta-analysis (**Figure 1**).

### Data extraction

First author name, country and year of publication, duration of the trial/weeks, duration of diabetes, age, gender, type and dose of GLP-1 agonists, total insulin dose in GLP-1 agonists and placebo arm, and the glycated hemoglobin in GLP-1 agonists and placebo were reported in tables and transferred manually to the RevMan, 5.4 for meta-analysis (**Tables 1**–**3**).

**Table 1:** Basic characteristics of the included patients.

Author	GLP-1 and dose	Country	Duration of study/weeks	Duration of diabetes/years	Age/years	Women%
Ahrén et al., 2016 (23)	Liraglutide all doses	USA	12–24 weeks	18.5 ± 2.5–24 ± 4.54	4.6 ± 2.5–44.8%	55.6%–62%
Dejgaard et al., 2024 (24)	Liraglutide. 1.8 mg	Denmark	12 weeks	18.9 ± 0.85	37.8	33.3
Frandsen et al., 2015 (25)	Liraglutide. 1.2 mg	Denmark	4 weeks	14.7 ± 9.96	18-50	50%
Ghanim et al., 2020 (26)	Liraglutide. 1.8 mg	Italy	52 weeks	Not mentioned	22.5	45.3%
Herold et al., 2020 (27)	Exenatide, 2 mg/W	Multi-center	26 weeks	21.05 ± 0.90	43.2	54%
Johansen et al., 2020 (28)	Exenatide, 10 microq/8h	Multi-center	26 weeks	21.05 ± 0.90	43.2	54%
Kielgast et al., 2011 (29)	Liraglutide. 1.2 mg	Multi-center	26 weeks	21.05 ± 0.90	43.2	54%
Kuhadiya et al., 2016 (30)	Liragutide, All doses	Belgium	52 weeks	21.4 ± 3.37	43.7	52.3%
Mathieu et al., 2016 (31)	Liraglutide, all doses	Denmark	24–52 weeks	19.6–22.5 ± 3.53	29 ± 6–50.3	53.6%–68.2%
Pozzilli et al., 2020 (32)	Albiglutide. 50 mg	Denmark	26 weeks	21.1	50.3	27.6%

**Table 2:** A comparison between GLP-1 agonists and placebo on the total daily insulin dose, and HbA1c among patients with type 1 diabetes mellitus.

Author	Insulin dose in GLP-1 agonists	Insulin dose in placebo	HbA1c reduction in GLP-1 agonists	HbA1c reduction in placebo	Total number of patients in GLP-1 agonists	The total number of patients in the placebo
Ahrén et al., 2016 (23)	Not assessed.	Not assessed.	0.27 ± 1.09	0.01 ± 0.37	628	206
Dejgaard et al., 2024 (24)	0.98 ± 0.145	0.14 ± 0.015	3.5 ± 0.50	3.5 ± 0.45	31	32
Frandsen et al., 2015 (25)	4.0 ± 1.3/20	0.0 ± 1.0/20	0.60 ± 0.20	0.50 ± 0.20	20	20
Ghanim et al., 2020 (26)	Not assessed	Not assessed	0.41 ± 0.18	0.29 ± 0.19	37	27
Herold et al., 2020 (27)	Not assessed	Not assessed	0.38 ± 0.98	0.19 ± 0.98	40	39
Johansen et al., 2020 (28)	7 ± 23.47	2 ± 23.41	0.30 ± 0.89	0.20 ± 0.79	54	54
Kielgast et al., 2011 (29), C-peptide +ve	0.19 ± 0.02	0.02± 0.01	0.20 ± 0.10	0.20 ± 0.10	8	8
Kielgast et al., 2011 (29), C peptide -ve	0.13 ± 0.02	0.20 ± 0.01	0.50 ± 0.10	0.20 ± 0.10	8	8
Kuhadiya et al., 2016 (30)	8.54 ± 4.63	1.9 ± 2.99	0.50 ± 0.64	0.30 ± 0.62	27	27
Mathieu et al., 2016 (31), all doses	No mean ± SD	No mean ± SD	0.49 ± 0.77	0.34 ± 0.80	1042	347
Pozzilli et al., 2020 (32) 1.2mg	0.04 ± 0.12	0.11 ± 0.2	0.59 ± 1.65	0.73 ± 1.03	12	43

**Table 3:** A comparison between liraglutide 0.6, 1.2, and 1.8 mg regarding the effects on total daily insulin dose, and HbA1c.

Character	Liraglutide 0.6 mg	Total patients	Liraglutide 1.2 mg	Total patients	Liraglutide 1.8 mg	Total patients
Total daily insulin dose reduction						
Ahrén et al., 2016 (17)	0.95 ± 0.05	211	0.93 ± 0.04	209	0.90 ± 0.05	205
Kuhadiya et al., 2016 (30)	2.8 ± 0.70	14	12.1 ± 0.70	16	10 ± 0.50	16
Mathieu et al., 2016 (31)	1.0 ± 0.04	350	0.95 ± 0.04	347	0.92 ± 0.04	347
HbA1c reduction						
Ahrén et al., 2016 (23)	0.24 ± 0.21	211	0.23 ± 0.21	209	0.35 ± 0.21	205
Kuhadiya et al., 2016 (30)	0.26 ± 0.17	14	0.78 ± 0.15	16	0.42 ± 0.15	16
Mathieu et al., 2016 (31)	0.09 ± 0.77	350	0.15 ± 0.12	347	0.20 ± 0.13	347

### Risk of bias assessment

We used the Cochrane Risk of Bias Assessment Tool (22), and all the included trials were of good quality. The Cochrane Risk of Bias evaluated the trials in terms of selection, performance, attrition, detection, and reporting bias (each component was evaluated as low-risk, high-risk of bias, and some concerns in **Table 4**.

**Table 4:** Risk of bias of the included studies.

Author	Random sequence generation bias	Allocation concealments bias	Blinding of participants and personnel	Blinding of outcome assessment	Incomplete outcome data	Selective reporting	Other bias
Ahrén et al., 2016 (23)	Low	Low	Low	Unclear	Low	Unclear	Low
Dejgaard et al., 2024 (24)	Low	Low	Low	Low	Low	Low	Low
Frandsen et al., 2015 (25)	Low	Low	Low	Low	Low	Low	Low
Ghanim et al., 2020 (26)	Low	Low	Low	High	High	High	High
Herold et al., 2020 (27)	Low	Low	Low	Unclear	Unclear	Low	Low
Johansen et al., 2020 (28)	Low	Low	Low	Low	Low	Low	Low
Kielgast et al., 2011 (29)	Low	Low	Low	Unclear	Unclear	Low	Low
Kuhadiya et al., 2016 (30)	Low	Low	Low	Unclear	Low	Low	Unclear
Mathieu et al., 2016 (31)	Low	Low	Unclear	Unclear	Unclear	Low	Unclear
Pozzilli et al., 2020 (32)	Low	Low	Low	Low	Low	Low	Low

### Data analysis

We used the Cochrane system for systematic Review (RevMan, version 5.4.1, Oxford, United Kingdom for data analysis, all the data were continuous, and the random effect was used due to the significant heterogeneity. The *I*^2^ was used to evaluate heterogeneity among studies (heterogeneity ≤25% was considered low, and ≥50% was considered substantial and the random effect was used). We investigated the effects of type and dose of GLP-1 agonists on HbA1c and the total daily insulin dose among patients with type 1 diabetes. The continuous data were evaluated as standard mean difference (SMD) using forest plots. Funnel plots were used in analysis with ≥10 studies for heterogeneity followed by sub-analysis to locate the source of heterogeneity (**Table 5**). The Chi-square, Z, and standard difference were estimated to assess the effects of GLP-1 agonists as additional therapy to insulin on HbA1c, and total daily insulin dose. We adopted a 95% confidence interval, and *P*-values of < 0.05 as significant.

**Table 5:** The effects of different studies on heterogeneity.

Author	Effect on heterogeneity
Ahrén et al., 2016 (23)	4% increase
Dejgaard et al., 2024 (24)	4% increase
Frandsen et al., 2015 (25)	3% increase
Ghanim et al., 2020 (26)	3% decrease
Herold et al., 2020 (27)	5% increase
Johansen et al., 2020 (28)	4% increase
Kielgast et al., 2011 (29)	44% decrease
Kuhadiya et al., 2016 (30)	5% increase
Mathieu et al., 2016 (31)	3% increase
Pozzilli et al., 2020 (32)	2% increase

## Results

### Characteristics of the included studies

The study included 10 trials, 6 were published in Europe, 3 were multi-nation, and one was from the United States of America, seven trials used liraglutide (doses 0.6 mg–1.8 mg/day), two studies used exenatide 10 microq/8 hourly, and 2 mg/week, and one used albiglutide 50 mg/day, the duration of the trials ranged from 4 to 52 weeks. The age of the patients ranged from 4.6 ± 2.5 to 50.3 years, females ranged from 27.6% to 68.2%, and the duration of T1DM ranged from 14.7 ± 9.96 to 24 ± 4.54 years. In the present meta-analysis, three trials compared different doses of liraglutide (0.6, 1.2, and 1.8 mg) (**Table 1**).

**Figure 2:**
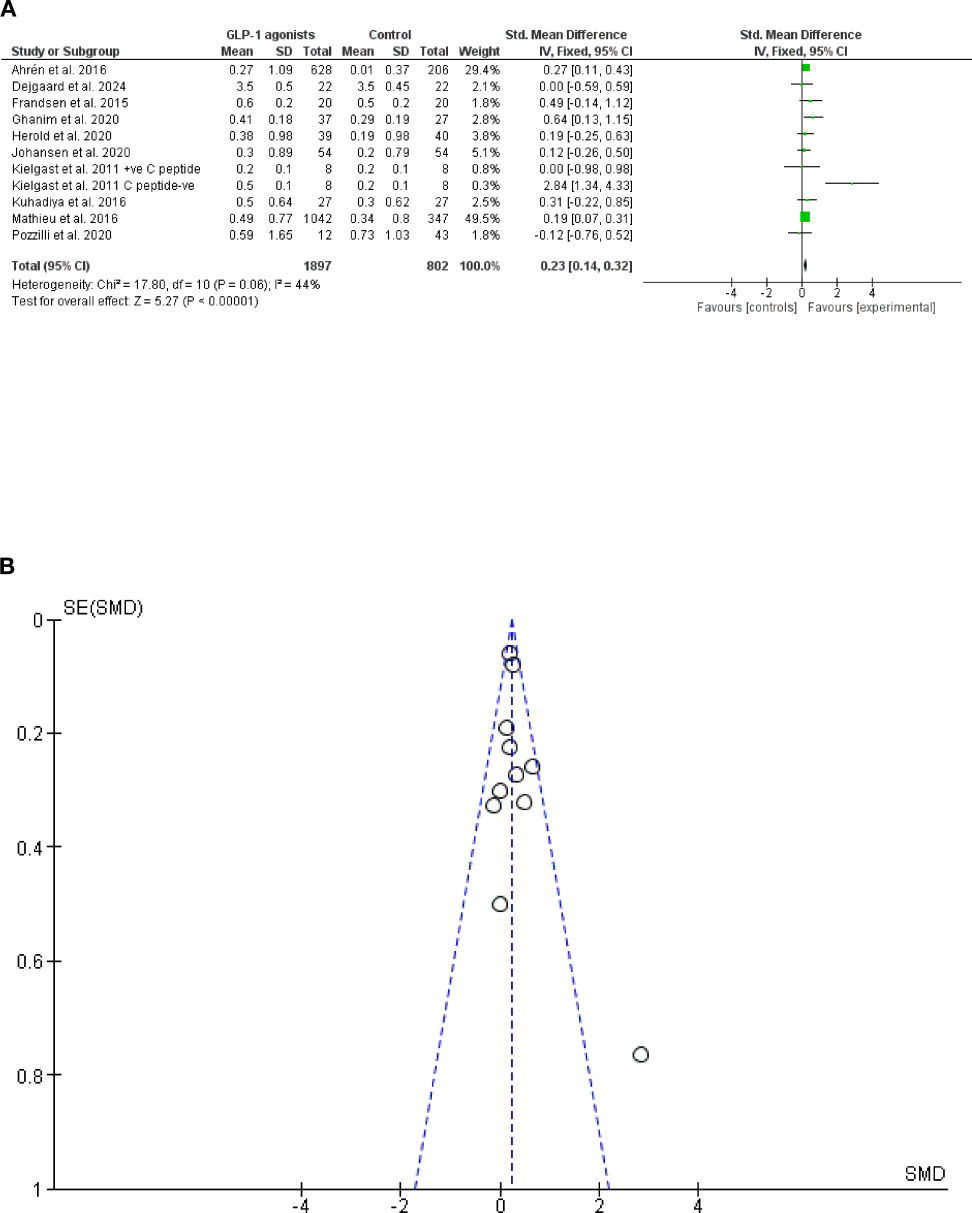
**(A)** HbA1c reduction following GLP-1 agonists among patients with type 1 diabetes mellitus forest plot. **(B)** HbA1c reduction following GLP-1 agonists among patients with type 1 diabetes mellitus Funnel plot.

In the present meta-analysis 11 cohorts from 10 trials (23–32) were pooled (2,699 patients were included). GLP-1 agonists are more effective than placebo in HbA1c reduction (7.47% vs. 6.46% reduction), *Z* = 5.27, SMD, 0.23, 95% CI (0.14–0.32), and *P*-value < 0.001, and the standard difference=10. However, a non-significant heterogeneity was found, chi-square, 17.80, *I*^2^ for heterogeneity = 44%, and *P*-value for heterogeneity, 0.06 (**Figure 2**).

**Figure 3:**
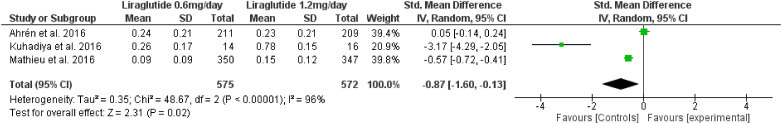
Liraglutide 0.6 mg versus 1.2 mg effects on HbA1c.

In a sub-analysis including three trials with 1,147 patients (23, 31, 32), we compared different doses of liraglutide. Liraglutide 1.2 mg was better than liraglutide 0.6 mg, *Z* = 2.31, SMD, −0.87, 95% CI (−1.60–0.13), *P*-value for overall effect, 0.02 with significant heterogeneity, chi-square, 48.67, and *I*^2^ for heterogeniety = 96%, and *P*-value for heterogeneity < 0.001 (**Figure 3**).

**Figure 4:**
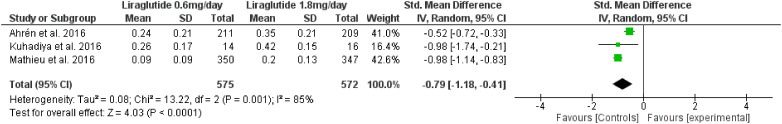
Liraglutide 0.6 mg versus 1.8 mg effects on HbA1c.

Similarly, liraglutide 1.8 mg was better than liraglutide 0.6, *Z* = 4.03, SMD, −0.79, 95% CI (−1.18–0.41), *P*-value for overall effect < 0.001 with significant heterogeneity, chi-square, 13.22, and *I*^2^ for heterogeneity = 88%, and *P*-value for heterogeneity, 0.001 (**Figure 4**).

**Figure 5:**
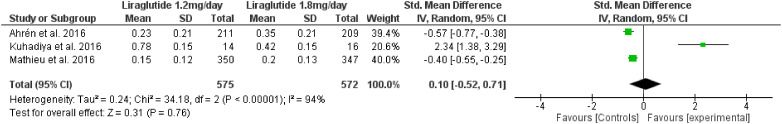
Liraglutide 1.2 mg versus 1.8 mg effects on HbA1c.

No significant differences between Liraglutide 1.2 mg and Liraglutide 1.8 mg regarding the effects on HbA1c, *Z* = 0.31, SMD, 0.10, 95% CI (−0.52–0.71), *P*-value for overall effect, 0.76 with significant heterogeneity, chi-square, 34.18, and *I*^2^ for heterogeneity = 94%, and *P*-value for heterogeneity < 0.001 (**Figure 5**).

**Figure 6:**
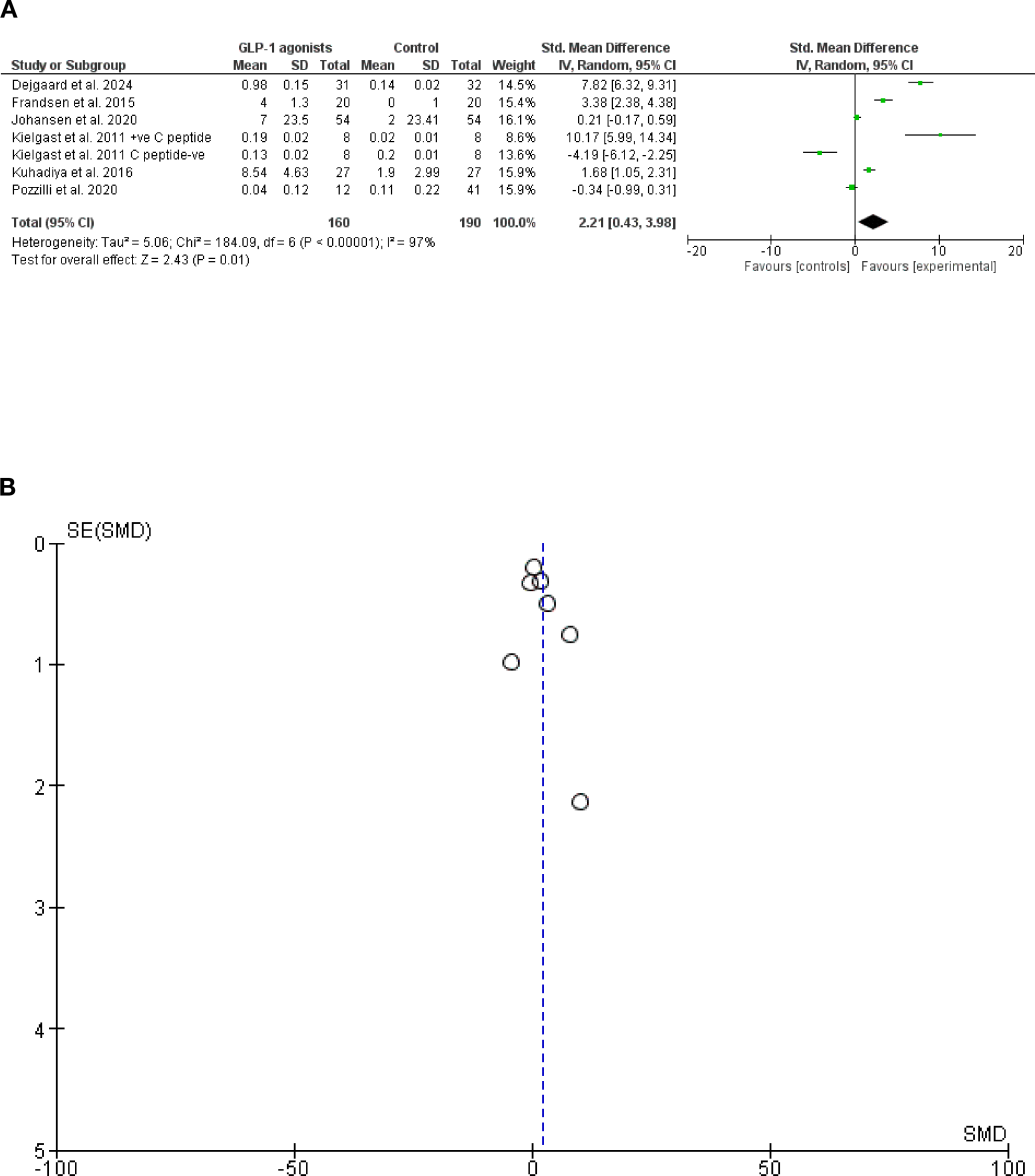
**(A)** Insulin dose following GLP-1 agonists among patients with type 1 diabetes mellitus forest plot. **(B)** Insulin dose following GLP-1 agonists among patients with type 1 diabetes mellitus Funnel plot.

Regarding insulin dose reduction, seven cohorts from six randomized trials (24, 25, 28–30, 32, 341 patients were included). GLP-1 agonists reduced total daily insulin dose, *Z* = 2.43, and *P*-value, 0.01, SMD, 2.21, 95% CI, (0.43–3.98), and the *SD* = 6. However, a significant heterogeneity was found, chi-square, 184.09, *I*^2^ for heterogeneity = 97%, and *P*-value for heterogeneity < 0.001 (**Figure 6**).

**Figure 7:**
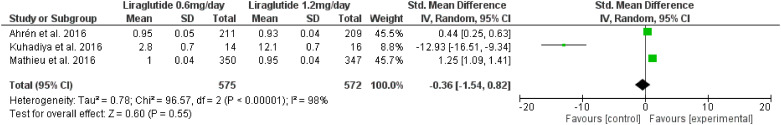
Liraglutide 0.6 mg versus 1.2 mg effects on total insulin dose.

A sub-analysis found no significant statistical difference between liraglutide 0.6mg and liraglutide 1.2 mg on total daily insulin dose, *Z* = 0.60, SMD, −0.36, 95% CI, −1.54–0.82, and *P*-value, 0.55, substantial heterogeneity was observed, found, chi-square, 96.57, *I*^2^ for heterogeneity = 98%, and *P*-value for heterogeneity < 0.001 (**Figure 7**).

**Figure 8:**
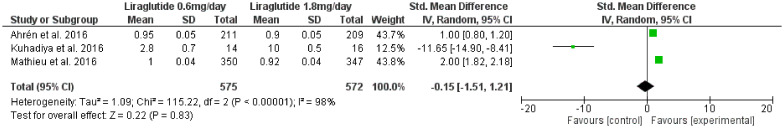
Liraglutide 0.6 mg versus 1.8 mg effects on total insulin dose.

Similarly, no significant difference was found when comparing liraglutide 0.6 mg and 1.8 mg, *Z* = 0.22, SMD, −0.15, 95% CI, −1.51–1.21, and *P*-value, 0.83, substantial heterogeneity was observed, chi-square, 115.22, *I*^2^ for heterogeneity = 98%, and *P*-value for heterogeneity < 0.001 (**Figure 8**).

## Discussion

GLP-1 agonists belong to the family of G proteins that couple with intracellular pathways to increase cyclic adenosine monophosphate to decrease glucagon release, enhance insulin secretion, enhance satiety, and decrease gastric emptying (33). The GLP-1 receptor agonists are synthetic incretins that are secreted by the gut endothelial cells in response to food; they enhance insulin secretion and decrease glucagon release to improve glycemic parameters and reduce body weight (34). However, the sustained pancreatic β-cell stimulation by GLP-1 agonists could exhaust them and lead to their failure (35).

In the present study, we found a reduction in the total daily insulin dose and HbA1c following the use of GLP-1 agonists as additional therapy to insulin, 2.21, 95% CI (0.43–3.98), and SMD, 0.23, 95% CI (0.14–0.32), respectively. Our findings are similar to Wang et al. (18), who included seven trials. However, Wang and colleagues included only seven studies; one of them added a monoclonal antibody that may significantly affect their findings due to the beta-cell preservation (36), and another included study is a poster presentation (37). In addition, they included studies published by the same authors. Furthermore, the authors included a study by Hari Kumar, which compared GLP-1 agonists and sitagliptin (38). Importantly, new studies were published after their study (25, 29). Our findings contradicted Kim et al. (19) findings, which included six trials; one of them assessed patients on a monoclonal antibody that affects the beta cells function and survival, and another included a study (20) that compared exenatide to DPP-4 inhibitors and not placebo. Karakasis et al. (20) included six trials. However, they focused on hypoglycemia and the time in the range.

Tan et al. (21) meta-analysis included ten trials to assess the glycated hemoglobin and five trials to assess the total daily insulin and found a benefit of GLP-1 agonists. However, he included studies published by the same authors, and some of the included studies were not found in the references list. This meta-analysis included the up-to-date and largest clinical trial and found a total daily insulin reduction and lowering effect on the glycated hemoglobin among patients with T1DM. The importance of this meta-analysis is that GLP-1 agonists are an effective add-on therapy to insulin among patients with T1DM.

T1DM treatment is solely based on multiple daily insulin injections, which are complex and are affected by patterns of meals and behavior with frequent dose adjustments. In addition, many patients with type 1 diabetes are not reaching glycemic targets. Therefore, they are prone to microvascular complications with significant physical and psychological consequences (33). Furthermore, insulin therapy is limited by hypoglycemia and weight gain, forcing some to reduce their insulin dose, leading to poor glycemic control (39). Another important issue is the needle phobia observed in a significant number of patients with diabetes (40). Previous studies on GLP-1 receptor agonists showed a reduction trend in the glycated hemoglobin improvement over time (1.7 reductions at 2 years, 1.6 at 5 years, and 1.5 at 7 years). Importantly, only 28% of the participants achieved an HbA1c of 7 at 3 years, and 46% achieved the same at 7 years. The higher good glycemic control at 7 years could be explained by the 72/194 drop-out rate at 3 years (41). β-cell exhaustion could be avoided by intensive lifestyle modification to reduce insulin requirement and beta cell stimulation (42). An important finding in this meta-analysis is the high efficacy of 1.2 mg liraglutide and 1.8 mg liraglutide over the 0.6 mg doses in reducing the glycated hemoglobin, SMD, −0.87, 95% CI (−1.60–0.13), and odd ratio, −0.79, 95% CI (−1.18–0.41), respectively. However, no significant differences were found between 1.2 mg and 1.8 mg doses, SMD, 0.10, 95% CI (−0.52–0.71). The above results imply that higher doses are more effective. In this meta-analysis, a significant reduction of total daily insulin dose was observed after GLP-1 agonists use, in similarity to Tan et al. (21), who found a total dose reduction, SMD, 1.99, 95% CI (0.52–3.46). However, Wang et al. observed no total daily insulin dose reduction in contradiction to the current findings (21). The contradiction in the findings could be due to not adjusting for insulin change per body weight. Our finding supported the Consensus Report by the American Diabetes Association (ADA) and the European Association for the Study of Diabetes that GLP-1 agonists may have roles in treating type 1 diabetes in particular patients with obesity and preserving beta-cell function (43). Interestingly, liraglutide 1.2 mg and 1.8 mg were not superior to liraglutide 0.6 mg, SMD, −0.36, 95% CI (−1.54–0.82), and SMD, −0.15, 95% CI (−1.51–1.21), respectively. This result should be viewed considering the possibility of statistical artifacts, the small size of the included trials, and the high heterogeneity. Park et al. (44) observed total insulin dose reduction with no effects on HbA1c; our results showed both total daily insulin dose and a lowering effect on HbA1c. Dimitrios et al. (45) concluded the beneficial effects of liraglutide on HbA1c and total daily insulin dose with liraglutide 1.8 mg being the most effective; their findings were similar to the current results. Importantly, our findings showed that both liraglutide 1.2 mg and liraglutide 1.8 mg are more effective than liraglutide 0.6 mg. Therefore, physicians may use liraglutide 1.2 mg to avoid unwanted gastrointestinal adverse events.

The current meta-analysis’s strengths is that this meta-analysis included the most up-to-date and largest clinical trial. The importance of this meta-analysis is that GLP-1 agonists are effective add-on therapy to insulin among patients with T1DM. We included randomized control trials and avoided the limitations of the previous meta-analyses (eliminating trials that compared GLP-1 to other antidiabetic medications and trials that included the addition of monoclonal antibodies). In addition, we excluded studies published by the same authors, including the more recent one.

### Study limitations

The study was limited by the high heterogeneity in the insulin dose arm, and we did not include the long-acting GLP-1 agonists (semaglutide and dulaglutide) because few studies assessed their role in T1DM.

### Conclusion

The above results showed that GLP-1 agonists are effective in reducing the total daily insulin dose and the glycated hemoglobin in T1DM. In addition, 1.2 mg and 1.8 mg liraglutide doses were more effective than 0.6 mg liraglutide doses. Physicians may need to titrate liraglutide from 0.6 mg to 1.2 mg and 1.8 mg to achieve better results. The above findings should be viewed in light of the small trials comparing the different doses of liraglutide, the high heterogeneity, and the possibility of statistical artifacts. Larger randomized controlled trials investigating the effects of the long-acting GLP-1 agonists at different doses are recommended. Studies addressing the effects of GLP-1 agonists on weight with a special focus on hypoglycemia and gastrointestinal side effects are needed.

## Data Availability

The original contributions presented in the study are included in the article/supplementary material. Further inquiries can be directed to the corresponding author.

## References

[1] Szadkowska A Michalak A Chylińska-Frątczak A Baranowska-Jaźwiecka A Koptas M Pietrzak I . Achieving target levels for vascular risk parameters in Polish school-age children with type 1 diabetes - a single center study. J Pediatr Endocrinol Metab. (2018) 31:1073–9. doi: 10.1515/jpem-2018-0098, PMID: 30240358

[2] Pasi R Ravi KS . Type 1 diabetes mellitus in pediatric age group: A rising endemic. J Family Med Prim Care. (2022) 11:27–31. doi: 10.4103/jfmpc.jfmpc_975_21, PMID: 35309606 PMC8930152

[3] Rosengren A Dikaiou P . Cardiovascular outcomes in type 1 and type 2 diabetes. Diabetologia. (2023) 66:425–37. doi: 10.1007/s00125-022-05857-5, PMID: 36640192 PMC9840171

[4] Mirghani H Alrasheed T Kalantan M Idris SM Adawi G . Diabulimia, the associations, and management: A narrative review. Tekyo Med J. (2022) 45:4549–54556.

[5] Tzogiou C Wieser S Eichler K Carlander M Djalali S Rosemann T . Incidence and costs of hypoglycemia in insulin-treated diabetes in Switzerland: A health-economic analysis. J Diabetes Complications. (2023) 37:108476. doi: 10.1016/j.jdiacomp.2023.108476, PMID: 37141836

[6] Steineck I Cederholm J Eliasson B Rawshani A Eeg-Olofsson K Svensson AM . Swedish National Diabetes Register. Insulin pump therapy, multiple daily injections, and cardiovascular mortality in 18,168 people with type 1 diabetes: observational study. BMJ. (2015) 350:h3234. doi: 10.1136/bmj.h3234, PMID: 26100640 PMC4476263

[7] Scheen AJ . Glucose-lowering agents and risk of ventricular arrhythmias and sudden cardiac death: A comprehensive review ranging from sulphonylureas to SGLT2 inhibitors. Diabetes Metab. (2022) 48:101405. doi: 10.1016/j.diabet.2022.101405, PMID: 36334794

[8] Guyton J Jeon M Brooks A . Glucagon-like peptide 1 receptor agonists in type 1 diabetes mellitus. Am J Health Syst Pharm. (2019) 76:1739–48. doi: 10.1093/ajhp/zxz179, PMID: 31612934

[9] von Herrath M Bain SC Bode B Clausen JO Coppieters K Gaysina L . ; Anti-IL-21–liraglutide Study Group investigators and contributors. Anti-interleukin-21 antibody and liraglutide for the preservation of β-cell function in adults with recent-onset type 1 diabetes: a randomised, double-blind, placebo-controlled, phase 2 trial. Lancet Diabetes Endocrinol. (2021) 9:212–24. doi: 10.1016/S2213-8587(21)00019-X, PMID: 33662334

[10] American Diabetes Association Professional Practice Committee . Diagnosis and classification of diabetes: standards of care in diabetes-2025. Diabetes Care. (2025) 48:S27–49. doi: 10.2337/dc25-S002, PMID: 39651986 PMC11635041

[11] Herman RJ Hayes MR Audrain-McGovern J Ashare RL Schmidt HD . Liraglutide attenuates nicotine self-administration as well as nicotine seeking and hyperphagia during withdrawal in male and female rats. Psychopharmacol (Berl). (2023) 240:1373–86. doi: 10.1007/s00213-023-06376-w, PMID: 37129617 PMC11088902

[12] Tuesta LM Chen Z Duncan A Fowler CD Ishikawa M Lee B . GLP-1 acts on habenular avoidance circuits to control nicotine intake. Nat Neurosci. (2017) 20:708–16. doi: 10.1038/nn.4540, PMID: 28368384 PMC5541856

[13] Malkin SJP Carvalho D Costa C Conde V Hunt B . The long-term cost-effectiveness of oral semaglutide versus empagliflozin and dulaglutide in Portugal. Diabetol Metab Syndr. (2022) 14:32. doi: 10.1186/s13098-022-00801-4, PMID: 35164855 PMC8845275

[14] Harris K Boland C Meade L Battise D . Adjunctive therapy for glucose control in patients with type 1 diabetes. Diabetes Metab Syndr Obes. (2018) 11:159–73. doi: 10.2147/DMSO.S141700, PMID: 29731652 PMC5927142

[15] Subramanian S Khan F Hirsch IB . New advances in type 1 diabetes. BMJ. (2024) 384:e075681. doi: 10.1136/bmj-2023-075681, PMID: 38278529

[16] Lewicka M Korzeniowska-Dyl I Moczulski D Woźniak-Kosek A Zawadzka M Henrykowska G . An analysis of epidemiological characteristics of microvascular complications and comorbidities among type 1 diabetes patients. Acta Biochim Pol. (2025) 72:14569. doi: 10.3389/abp.2025.14569, PMID: 40475072 PMC12137067

[17] Prinz N Tittel SR Bachran R Birnbacher R Brückel J Dunstheimer D . Characteristics of patients with type 1 diabetes and additional autoimmune disease in the DPV registry. J Clin Endocrinol Metab. (2021) 106:e3381–9. doi: 10.1210/clinem/dgab376, PMID: 34061946

[18] Wang W Liu H Xiao S Liu S Li X Yu P . Effects of insulin plus glucagon-like peptide-1 receptor agonists (GLP-1RAs) in treating type 1 diabetes mellitus: A systematic review and meta-analysis. Diabetes Ther. (2017) 8:727–38. doi: 10.1007/s13300-017-0282-3, PMID: 28616805 PMC5544618

[19] Kim YJ Hwang SD Lim S . Effects of sodium-glucose cotransporter inhibitor/glucagon-like peptide-1 receptor agonist add-on to insulin therapy on glucose homeostasis and body weight in patients with type 1 diabetes: A network meta-analysis. Front Endocrinol (Lausanne). (2020) 11:553. doi: 10.3389/fendo.2020.00553, PMID: 32973680 PMC7466678

[20] Karakasis P Koufakis T Patoulias D Barkas F Klisic A Mitrovic M . Effects of glucagon-like peptide-1 receptor agonists on glycated haemoglobin and continuous glucose monitoring metrics as adjunctive therapy to insulin in adults with type 1 diabetes: A meta-analysis of randomized controlled trials. Diabetes Obes Metab. (2024) 26:6043–54. doi: 10.1111/dom.15979, PMID: 39344842

[21] Tan X Pan X Wu X Zheng S Chen Y Liu D . Glucagon-like peptide-1 receptor agonists as add-on therapy to insulin for type 1 diabetes mellitus. Front Pharmacol. (2023) 14:975880. doi: 10.3389/fphar.2023.975880, PMID: 38249345 PMC10797415

[22] Yang ZR Sun F Zhan SY . Risk on bias assessment: (2) Revised Cochrane risk of bias tool for individually randomized, parallel group trials (RoB2.0). Zhonghua Liu Xing Bing Xue Za Zhi. (2017) 38:1285–91. doi: 10.3760/cma.j.issn.0254-6450.2017.09.028, PMID: 28910948

[23] Ahrén B Hirsch IB Pieber TR Mathieu C Gómez-Peralta F Hansen TK . Efficacy and safety of liraglutide added to capped insulin treatment in subjects with type 1 diabetes: the ADJUNCT TWO randomized trial. Diabetes Care. (2016) 39:1693–701. doi: 10.2337/dc16-0690, PMID: 27493132

[24] Dejgaard TF Frandsen CS Kielgast U Størling J Overgaard AJ Svane MS . Liraglutide enhances insulin secretion and prolongs the remission period in adults with newly diagnosed type 1 diabetes (the NewLira study): A randomized, double-blind, placebo-controlled trial. Diabetes Obes Metab. (2024) 26:4905–15. doi: 10.1111/dom.15889.normalweight, PMID: 39192527

[25] Frandsen CS Dejgaard TF Holst JJ Andersen HU Thorsteinsson B Madsbad S . Twelve-week treatment with liraglutide as add-on to insulin in normal-weight patients with poorly controlled type 1 diabetes: A randomized, placebo-controlled, double-blind parallel study. Diabetes Care. (2015) 38:2250–7. doi: 10.2337/dc15-1037.normalweight, PMID: 26486191

[26] Ghanim H Batra M Green K Abuaysheh S Hejna J Makdissi A . Liraglutide treatment in overweight and obese patients with type 1 diabetes: A 26-week randomized controlled trial; mechanisms of weight loss. Diabetes Obes Metab. (2020) 22:1742–52. doi: 10.1111/dom.14090, PMID: 32424935

[27] Herold KC Reynolds J Dziura J Baidal D Gaglia J Gitelman SE . Exenatide extended release in patients with type 1 diabetes with and without residual insulin production. Diabetes Obes Metab. (2020) 22:2045–54. doi: 10.1111/dom.14121, PMID: 32573927 PMC8009602

[28] Johansen NJ Dejgaard TF Lund A Schlüntz C Hartmann B Holst JJ . Effects of short-acting exenatide added three times daily to insulin therapy on bone metabolism in type 1 diabetes. Diabetes Obes Metab. (2022) 24:221–7. doi: 10.1111/dom.14568.normalweight, PMID: 34617375

[29] Kielgast U Krarup T Holst JJ Madsbad S . Four weeks of treatment with liraglutide reduces insulin dose without loss of glycemic control in type 1 diabetic patients with and without residual beta-cell function. Diabetes Care. (2011) 34:1463–8. doi: 10.2337/dc11-0096.normalweight, PMID: 21593296 PMC3120168

[30] Kuhadiya ND Dhindsa S Ghanim H Mehta A Makdissi A Batra M . Addition of liraglutide to insulin in patients with type 1 diabetes: A randomized placebo-controlled clinical trial of 12 weeks. Diabetes Care. (2016) 39:1027–35. doi: 10.2337/dc15-1136, PMID: 27208343 PMC5864130

[31] Mathieu C Zinman B Hemmingsson JU Woo V Colman P Christiansen E . Efficacy and safety of liraglutide added to insulin treatment in type 1 diabetes: the ADJUNCT ONE treat-to-target randomized trial. Diabetes Care. (2016) 39:1702–10. doi: 10.2337/dc16-0691, PMID: 27506222

[32] Pozzilli P Bosi E Cirkel D Harris J Leech N Tinahones FJ . Randomized 52-week phase 2 trial of albiglutide versus placebo in adult patients with newly diagnosed type 1 diabetes. J Clin Endocrinol Metab. (2020) 105:dgaa149. doi: 10.1210/clinem/dgaa149.normalweight, PMID: 32219329

[33] Krashes M . Glucagon-like peptide-1 receptor. Curr Biol. (2024) 34:R1163–4. doi: 10.1016/j.cub.2024.10.039, PMID: 39626623

[34] Janket SJ Chatanaka MK Sohaei D Tamimi F Meurman JH Diamandis EP . Does incretin agonism have sustainable efficacy? Cells. (2024) 13:1842. doi: 10.3390/cells13221842, PMID: 39594592 PMC11592889

[35] Philis-Tsimikas A Wysham CH Hardy E Han J Iqbal N . Efficacy and tolerability of exenatide once weekly over 7 years in patients with type 2 diabetes: An open-label extension of the DURATION-1 study. J Diabetes Complicat. (2019) 33:223–30. doi: 10.1016/j.jdiacomp.2018.11.012, PMID: 30600137

[36] Sarkar G Alattar M Brown RJ Quon MJ Harlan DM Rother KI . Exenatide treatment for 6 months improves insulin sensitivity in adults with type 1 diabetes. Diabetes Care. (2014) 37:666–70. doi: 10.2337/dc13-1473, PMID: 24194508 PMC3931382

[37] Hamamoto Y Mori K Honjo S Kawasaki Y Fujimoto\ K Tatsuoka H . One-year effects of liraglutide on pancreatic beta cell function and glycemic control in Japanese type 1 diabetes with residual insulin secretion. Diabetologia. (2012) 55:S300.

[38] Hari Kumar KV Shaikh A Prusty P . Addition of exenatide or sitagliptin to insulin in new onset type 1 diabetes: a randomized, open label study. Diabetes Res Clin Pract. (2013) 100:e55–8. doi: 10.1016/j.diabres.2013.01.020, PMID: 23490599

[39] Duncanson E Le Leu RK Shanahan L Macauley L Bennett PN Weichula R . The prevalence and evidence-based management of needle fear in adults with chronic disease: A scoping review. PloS One. (2021) 16:e0253048. doi: 10.1371/journal.pone.0253048, PMID: 34111207 PMC8192004

[40] Almohareb SN Alfayez OM Aljuaid SS Alshahrani WA Bakhsh G Alshammari MK . Effectiveness and safety of GLP-1 receptor agonists in patients with type 1 diabetes. J Clin Med. (2024) 13:6532. doi: 10.3390/jcm13216532, PMID: 39518671 PMC11546400

[41] Page KA Reisman T . Interventions to preserve beta-cell function in the management and prevention of type 2 diabetes. Curr Diabetes Rep. (2013) 13:252–60. doi: 10.1007/s11892-013-0363-2, PMID: 23371283 PMC3595345

[42] Holt RIG DeVries JH Hess-Fischl A Hirsch IB Kirkman MS Klupa T . The management of type 1 diabetes in adults. A consensus report by the american diabetes association (ADA) and the european association for the study of diabetes (EASD). Diabetes Care. (2021) 44:2589–625. doi: 10.2337/dci21-0043, PMID: 34593612

[43] Irfan H Pallipamu N Farhat H Gutlapalli SD Thiagaraj SS Shukla TS . Role of Glucagon-Like Peptide-1 Receptor Agonists on the Weight Loss of Individuals With Type 2 Diabetes: A Systematic Review only for discussion. Cureus. (2023) 15:e40448. doi: 10.7759/cureus.40448, PMID: 37456411 PMC10349654

[44] Park J Ntelis S Yunasan E Downton KD Yip TC Munir KM . Glucagon-like peptide 1 analogues as adjunctive therapy for patients with type 1 diabetes: an updated systematic review and meta-analysis. J Clin Endocrinol Metab. (2023) 109:279–92. doi: 10.1210/clinem/dgad471, PMID: 37561012

[45] Dimitrios P Michael D Vasilios K Konstantinos S Konstantinos I Ioanna Z . Liraglutide as adjunct to insulin treatment in patients with type 1 diabetes: A systematic review and meta-analysis. Curr Diabetes Rev. (2020) 16:313–26. doi: 10.2174/1573399815666190614141918, PMID: 31203802

